# Anticancer, Anti-Inflammatory, and Analgesic Activities of Synthesized 2-(Substituted phenoxy) Acetamide Derivatives

**DOI:** 10.1155/2014/386473

**Published:** 2014-08-14

**Authors:** Priyanka Rani, Dilipkumar Pal, Rahul Rama Hegde, Syed Riaz Hashim

**Affiliations:** ^1^Department of Chemistry, School of Sciences, IFTM University, Moradabad, Uttar Pradesh, India; ^2^Department of Pharmaceutical Sciences, Guru Ghasidas Vishwavidyalaya (A Central University), Koni, Bilaspur, Chhattisgarh 495 009, India; ^3^Department of Pharmaceutics, School of Pharmaceutical Sciences, IFTM University, Moradabad, Uttar Pradesh, India; ^4^Department of Chemistry, School of Pharmaceutical Sciences, IFTM University, Moradabad, Uttar Pradesh, India

## Abstract

The aphorism was to develop new chemical entities as potential anticancer, anti-inflammatory, and analgesic agents. The Leuckart synthetic pathway was utilized in development of novel series of 2-(substituted phenoxy)-N-(1-phenylethyl)acetamide derivatives. The compounds containing 1-phenylethylamine as basic moiety attached to substituted phenols were assessed for their anticancer activity against MCF-7 (breast cancer), SK-N-SH (neuroblastoma), anti-inflammatory activity, and analgesic activity. These investigations revealed that synthesized products **3a–j** with halogens on the aromatic ring favors as the anticancer and anti-inflammatory activity. Among all, compound **3c** N-(1-(4-chlorophenyl)ethyl)-2-(4-nitrophenoxy)acetamide exhibited anticancer, anti-inflammatory, and analgesic activities. In conclusion, **3c** may have potential to be developed into a therapeutic agent.

## 1. Introduction

In the past decade, numerous advances have taken place in the understanding of pathogenesis of cancer and its relationship with inflammation. To date the research work on this ground is substantial, but it still lacks clinical accomplishment, with the existing development facade over the clinical exploitation of drugs with dual acting cyclooxygenase-2 (COX-2) inhibition and antiproliferative potency [1]. Whenever cells in the body are allowed to divide uncontrollably and to metastasize, this results in the formation of cancer. Large scale of studies indicates that overexpression of COX-2 strongly appeared in human breast carcinomas approximately 40% and in colorectal carcinomas approximately 60%. In particular, COX-2 is found during overexpression of human epidermal growth factor receptor 2 (HER2/neu). The amplification of this oncogene plays an important role in the development of some aggressive types of breast cancer. COX-2 is also been reported in early tumors by stromal cells [2] and in larger tumors by dysplastic epithelium [3]. It has been proved that various inflammatory cells, cytokines, chemokines, and enzymes facilitate the development of cancers from inflammation [4]. A few COX-2/LOX-2 (lipooxygenase-2) inhibitors such as sulindac, celecoxib, licofelone, and aspirin analogues have also been reported as suppressors of malignant growth of cells* in vitro* and* in vivo* [5] and Alzheimer's disease [6]. This designates that there is an unfamiliar association involving cancer and inflammation [7].

Nowadays no data are available concerning the potential use of anticancer agents as COX/LOX inhibitors. Recent molecular targets for the treatment of cancer are the relation between arachidonic acid (AA) and carcinogenesis because of the regulation of AA by two enzymes, cyclooxygenases (COXs) and lipoxygenases (LOXs). Prostaglandin E_2_ (PGE_2_), the main product of COX-2, is found in high concentration in tumor cells [8] and is synthesized by various human breast cancer cell lines. The goal of this research was to achieve a novel series of agents that could have potent anticancer efficacy in association with the suppression of COX/LOX pathways.

**Figure 1:**
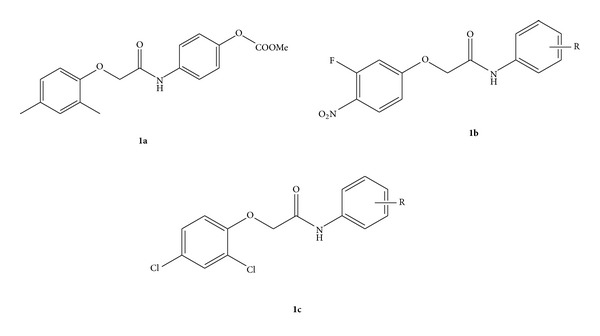
The commonly used structures (**1a**,** 1b** 2-Phenoxy-N-phenylacetamide core nucleus with antimycobacterial activity) (**1c** phenoxy-N-phenylacetamide compounds with potent P-gp inhibitor activities).

In the present paper a novel and efficient strategy has been developed to synthesize N-(1-(4-chlorophenyl) ethyl)-2-(substituted phenoxy)acetamide derivatives and 2-(substituted phenoxy)-N-(1-(p-tolyl)ethyl)acetamide derivatives with excellent yields. This synthetic pathway was established by the Leuckart reaction. The Leuckart reaction is a process for the reductive amination of aldehydes and ketones by formamide, ammonium formate, or formic acid with formamide [9]. This reaction has been used for the aromatic compounds, but very little work has been reported for the synthesis of aliphatic compounds (synthesis of 2-heptylamine, propylamine, and isopropylamine) [10]. Apart from the aliphatic compounds, 1,4-benzodiazepines were synthesized by the Leuckart Wallach reaction containing the benzodiazepine scaffold and were originated with various biological activities (benzodiazepine family) comprised mainly of central nervous system (CNS) suppressant due to its anxiolytic, anticonvulsant, sedative, and muscle relaxant activities. It is used in various marketed drugs such as alprazolam, bromazepam, chlorazepate, and valium [11]. 1,4-Benzodiazepines also demonstrate therapeutic activities and are used as antibiotics [12], antiulcers [13], and anti-HIV agents [14]. They are also used as farnesyltransferase inhibitors [15]. Compounds with 2-phenoxy-N-phenylacetamide core nucleus as in Figure 1 (**1a** and** 1b**) have shown remarkable research and demonstrated a variety of biological activities such as antimycobacterial [16], antiparasitic [17], antiviral [18], and anticancer [19]activities [20–23]. These compounds are also reported with inhibition of Pgp efflux transporters which are beneficial in the treatment of multidrug resistant strains of cancer cells. The phenoxy-N-phenylacetamide compounds are reported to be less toxic and more effective as that of potent P-gp inhibitors (coumarin analogues, 2-adamantyl analogues); see Figure 1 (**1c**) [24]. Now, we are reporting a new series of agents that have potent anticancer activities. These agents possibly may act upon COX/LOX pathways which may be included as a part of our future research program.

These compounds were further biologically evaluated for analgesic activity, and few of them showed activity. For the development of novel anticancer agents with lower toxic effect and higher efficiency, we carried out high throughput screening, which resulted in the discovery of titled compounds. Now, we report structure activity relationships and biological assessment of the titled compounds.

## 2. Experiment

### 2.1. General Considerations

All research chemicals were purchased from CDH (Central Drug House P. Ltd., New Delhi, India) and used as such for the reactions. These were used without further purification. Purification of the synthesized compounds was carried out by the recrystallization with appropriate solvent in case of solids but by distillation in case of liquids. Purity of the compounds and completion of reactions were monitored by thin layer chromatography (TLC) using silica gel G as the stationary phase and mobile phases used were n-hexane  :  ethyl acetate (1 : 1). Spots were visualized by exposure to iodine vapour. Melting points were determined in open capillaries on Thomas Hoover apparatus and are uncorrected. IR spectra were recorded on a Shimadzu IR-435 spectrophotometer using KBr pellets and 1H-NMR spectra were recorded on a Bruker 400 MHz spectrometer (Bruker Corporation, Massachusetts, USA) instrument using tetramethylsilane (TMS) as an internal standard and DMSO-d6 as a solvent. Mass spectra were recorded on Micromass Q-Tof Micro (Waters Corporation Massachusetts, USA). Chemical shifts are given in parts per million (ppm). The anticancer activity was carried out at Tata Memorial Hospital, Mumbai. The anti-inflammatory and analgesic screenings are carried out at pharmacology laboratory of College of Pharmacy IFTM University, Moradabad. The anti-inflammatory activity was carried out using digital plethysmometer (Orchid Scientific, Maharashtra, India). The analgesic activity was carried out by Eddy hot plate method using analgesiometer (Sanghmeshwar International, Ambala, India). All the animal experiments were approved by Institutional Animal Ethical Committee (IAEC).

### 2.2. Synthesis

#### 2.2.1. General Method for the Synthesis of 1-(4-Chlorophenyl)ethanamine ** (1a) ** and 1-(p-Tolyl)ethanamine ** (1b) ** from 1-(4-Chlorophenyl)ethanone and 1-(p-Tolyl)ethanone, Respectively

This procedure of the Leuckart reaction was used for the preparation of amines from ketones. Ammonium carbonate (215 gm, 4 mols) was placed in a 1 litre three-necked round bottom flask, which was fitted with a thermometer, a dropping funnel, and a bent tube attached for distillation to a short condenser. Formic acid (98%, 109 mL) was taken in the dropping funnel and added dropwise. When the reaction subsided, the mixture was heated slowly until the temperature of the reaction increased to about 165°C. The ketone (1 mol) was added in one lot and the temperature was slowly raised to 180–185°C. Ammonia, water, carbon dioxide, and some of the unreacted ketones distilled over. The distilled ketone was separated and returned to the reaction mixture. The mixture which gradually became homogenous was maintained at 180–185°C for 4-5 hours. When the reaction was complete, the mixture was cooled and stirred thoroughly with twice its volume of water. The aqueous layer was separated and the formyl derivative of the amine (nonaqueous layer) so obtained was refluxed with 100–150 mL of concentrated hydrochloric acid for 2-3 hours. After the hydrolysis, the reaction mixture was cooled and extracted with ether to remove any unreacted ketone. The aqueous solution was made strongly alkaline with 30% sodium hydroxide solution and the separated amine was extracted with ether. The ethereal extract was dried over anhydrous sodium sulphate, and after removal of the solvent, the product distilled under reduced pressure.

#### 2.2.2. General Method for the Synthesis of 2-Chloro-N-(1-(4-chlorophenyl)ethyl)acetamide ** (2a) ** and 2-Chloro-N-(1-(p-tolyl)ethyl)acetamide **(2b) ** from ** (1a) ** and ** (1b)**, Respectively

An ice-cooled aqueous solution of sodium hydroxide (50 mL, 10%) was taken in two different well-corked conical flasks; then, 0.1 mol of synthesized compounds** 1a** and** 1b** was added in both the flasks very slowly followed by addition of chloroacetyl chloride (11.93 mL, 0.15 mol) dropwise with constant stirring and shaking. The reaction was strongly shaken until odor of chloroacetyl chloride moved out. The pH of reaction mixture was kept around 9-10 by the addition of sodium hydroxide solution. Filter off the product and solid amides** 2a** and** 2b** that formed were washed thoroughly with water, dried, and recrystallized from ethanol.

#### 2.2.3. General Method for the Synthesis of N-(1-(4-Chlorophenyl)ethyl)-2-(substituted phenoxy)acetamide (**3a–e**) and 2-(Substituted phenoxy)-N-(1-(p-tolyl)ethyl)acetamide (**3f–j**) Derivatives from (**2a**) and (**2b**), Respectively

Phenoxy acetamide derivatives were prepared by reacting (**2a-2b**) (0.01 mol) with different substituted phenols (0.01 mol) in presence of anhydrous potassium carbonate (0.01 mol) and catalytic amount of potassium iodide in refluxing dry acetone. In some cases unreacted phenol was removed from the final product by treating the substance with 10% w/v sodium carbonate solution in water. The compound was then filtered and washed thoroughly with water and recrystallised from appropriate solvent. The completion of the reaction was monitored by TLC.

*(1) N-(1-(4-Chlorophenyl)ethyl)-2-phenoxyacetamide (* **3a** *).* IR (KBr, cm^−1^). 3205, 3112, 3014, 2928, 1711, 1644, 1318, 1235. ^1^H NMR (400 MHz, DMSO-d_6_, *δ* ppm): 8.15 (s, 1H, NH), 7.52–6.90 (m, 4H, H-2′, H-3′, H-5′, H-6′), 6.81 (t, 2H, H-3′′, H-5′′), 6.20–5–93 (m, 3H, H-2′′, H-4′′, H-6′′), 5.80–5.47 (m, 1H, CH-3), 5.21 (s, 2H, CH_2_-2), 1.74 (d, 3H, CH_3_-4). ^13^C NMR (DMSO-d_6_, *δ* ppm): 166.4 (C=O, NHCO), 159.8 (C, C-1′′), 141.7 (C, C-1′), 135.4 (C, C-4′), 130.1 (CH, C-3′′, 5′′), 125.1 (CH, C-3′, 5′), 124.4 (CH, C-2′, 6′), 120.8 (CH, C-4′′), 116.1 (CH, C-2′′, 6′′), 68.4 (CH_2_, C-2), 55.9 (CH, C-3), 25.9 (CH_3_, C-4). mass: m/z 289 (M^+^), 290 (M+1, 17.5%), 291 (M+2, 33.4%). Anal. Calc. For C_16_H_16_ClNO_2_: C 66.32, H 5.57, N 4.83. Found: C 66.28, H 5.53, N 4.85. 

*(2) 2-(4-Bromophenoxy)-N-(1-(4-chlorophenyl)ethyl)acetamide (* **3b** *).* IR (KBr, cm^−1^): 3245, 3056, 3017, 2915, 1678, 1622, 1335, 1105. ^1^H NMR (400 MHz, DMSO-d_6_, *δ* ppm): 8.25 (s, 1H, NH), 7.81–7.12 (m, 6H, H-2′, H-3′, H-5′, H-6′, H-3′′, H-5′′), 6.80 (d, 2H, H-2′′, H-6′′), 6.31–6.24 (m, 1H, CH-3), 5.34 (s, 2H, CH_2_-2), 1.63 (d, 3H, CH_3_-4). ^13^C NMR (DMSO-d_6_, *δ* ppm): 170.1 (C=O, NHCO), 162.5 (C, C-1′′), 142.4 (C, C-1′), 139.8 (C, C-4′), 138.7 (CH, C-3′′, 5′′), 127.2 (CH, C-3′, 5′), 123.8 (CH, C-2′, 6′), 120.8 (CH, C-2′′, 6′′), 111.9 (C, C-4′′), 66.7 (CH_2_, C-2), 50.9 (CH, C-3), 23.8 (CH_3_, C-4). mass: m/z 369 (M^+^), 367 (M-2, 76.5%), 371 (M+2, 25.3%). Anal. Calc. For C_16_H_15_BrClNO_2_: C 52.13, H 4.10, N 3.80. Found: C 52.09, H 4.13, N 3.77. 

*(3) N-(1-(4-Chlorophenyl)ethyl)-2-(4-nitrophenoxy)acetamide (* **3c** *).* IR (KBr, cm^−1^): 3255, 3218, 3122, 2817, 1686, 1533, 1528, 1213, 1110. ^1^H NMR (400 MHz, DMSO-d_6_, *δ* ppm): 8.25 (d, 2H, H-3′′, H-5′′), 7.92 (s, 1H, NH), 7.81–7.35 (m, 4H, H-2′, H-3′, H-5′, H-6′), 7.17 (d, 2H, H-2′′, H-6′′), 5.78–5.31 (m, 1H, CH-3), 4.92 (s, 2H, CH_2_-2), 2.11 (d, 3H, CH_3_-4). ^13^C NMR (DMSO-d_6_, *δ* ppm): 170.5 (C=O, NHCO), 163.7 (C, C-1′′), 148.4 (C, C-4′′), 145.5 (C, C-1′), 139.1 (C, C-4′), 132.4 (CH, C-3′, 5′), 130.4 (CH, C-2′, 6′), 129.7 (CH, C-3′′, 5′′), 120.7 (CH, C-2′′, 6′′), 71.4 (CH_2_, C-2), 69.1 (CH, C-3), 35.4 (CH_3_, C-4). mass: m/z 334 (M^+^), 335 (M+1, 18.7%), 336 (M+2, 34.1%). Anal. Cacl. For C_16_H_15_ClN_2_O_4_: C 57.41, H 4.52, N 8.37. Found: C 57.45, H 4.48, N 8.32. 

*(4) 2-(4-(Tert-butyl)phenoxy)-N-(1-(4-chlorophenyl)ethyl)acetamide (* **3d** *).* IR (KBr, cm^−1^): 3217, 3181, 2973, 2810, 1688, 1611, 1290, 1176. ^1^H NMR (400 MHz, DMSO-d_6_, *δ* ppm): 8.22 (s, 1H, NH), 8.10–7.37 (m, 6H, H-2′, H-3′, H-5′, H-6′, H-3′′, H-5′′), 7.21 (d, 2H, H-2′′, H-6′′), 5.20–5.13 (m, 1H, CH-3), 4.94 (s, 2H, CH_2_-2), 3.11 (d, 3H, CH_3_-4), 2.83 (s, 9H, (CH_3_)_3_). ^13^C NMR (DMSO-d_6_, *δ* ppm): 169.8 (C=O, NHCO), 159.4 (C, C-1′′), 151.8 (C, C-4′′), 137.8 (C, C-1′), 131.8 (C, C-4′), 130.4 (CH, C-3′, 5′), 128.1 (CH, C-2′, 6′), 123.4 (CH, C-3′′, 5′′), 110.4 (CH, C-2′′, 6′′), 64.5 (CH_2_, C-2), 61.8 (CH, C-3), 41.4 (C, C-(CH_3_)_3_), 40.9 ((CH_3_)_3_), 19.7 (CH_3_, C-4). mass: m/z 345 (M^+^), 346 (M+1, 22.3%), 347 (M+2, 32.4%). Anal. Calc. For C_20_H_24_ClNO_2_: C 69.45, H 6.99, N 4.05. Found: C 69.41, H 6.94, N 4.10. 

*(5) N-(1-(4-Chlorophenyl)ethyl)-2-(4-methoxyphenoxy)acetamide (* **3e** *).* IR (KBr, cm^−1^): 3216, 3122, 2988, 2917, 2713, 1677, 1609, 1200, 1174. ^1^H NMR (400 MHz, DMSO-d_6_, *δ* ppm): 8.67 (s, 1H, NH), 7.94–7.36 (m, 4H, H-2′, H-3′, H-5′, H-6′), 7.20 (s, 4H, H-2′′, H-3′′, H-5′′, H-6′′), 5.43–5.21 (m, 1H, CH-3), 5.11 (s, 2H, CH_2_-2), 4.64 (s, 3H, OCH_3_), 2.36 (d, 3H, CH_3_-4). ^13^C NMR (DMSO-d_6_, *δ* ppm): 172.9 (C=O, NHCO), 154.2 (C, C-4′′), 152.8 (C, C-1′′), 139.8 (C, C-1′), 137.2 (C, C-4′), 131.2 (CH, C-3′, 5′), 130.1 (CH, C-2′, 6′), 121.1 (CH, 2′′, 3′′, 5′′, 6′′), 73.3 (CH_2_, C-2), 61.4 (CH_3_, OCH_3_), 60.2 (CH, C-3), 30.1 (CH_3_, C-4). mass: m/z 319 (M^+^), 320 (M+1, 19.6%), 321 (M+2, 34.1%). Anal. Calc. For: C_17_H_18_ClNO_3_: C 63.85, H 5.67, N 4.38. Found: C 63.81, H 5.70, N 4.32. 

*(6) 2-Phenoxy-N-(1-(p-tolyl)ethyl)acetamide (* **3f** *).* IR (KBr, cm^−1^): 3266, 3033, 2895, 1654, 1622, 1278, 1112.^1^H NMR (400 MHz, DMSO-d_6_, *δ* ppm): 8.32 (s, 1H, NH), 8.11 (t, 2H, H-3′′, H-5′′), 7.46–7.31 (m, 4H, H-2′, H-3′, H-5′, H-6′), 7.12–6.93 (m, 3H, H-2′′, H-4′′, H-6′′), 6.81-6.78 (m, 1H, CH-3), 6.61 (s, 2H, CH_2_-2), 3.36 (s, 3H, CH_3_-4′), 2.45 (d, 3H, CH_3_-4). ^13^C NMR (DMSO-d_6_, *δ* ppm): 171.8 (C=O, NHCO), 167.4 (C, C-1′′), 151.2 (C, C-1′), 150.1 (C, C-4′), 132.4 (CH, C-3′′, 5′′), 130.6 (CH, C-3′, 5′), 129.8 (CH, C-2′, 6′), 127.1 (CH, C-4′′), 122.7 (CH, C-2′′, 6′′), 66.8 (CH_2_, C-2), 57.1 (CH, C-3), 20.9 (CH_3_, C-4), 18.7 (CH_3_, CH_3_-C4′). mass: m/z 269 (M^+^), 270 (M+1, 19.6%), 271 (M+2, 2.7%). Anal. Calc. For C_17_H_19_NO_2_: C 75.81, H 7.11, N 5.20. Found: C 75.84, H 7.05, N 5.24. 

*(7) 2-(4-Bromophenoxy)-N-(1-(p-tolyl)ethyl)acetamide (* **3g** *).* IR (KBr, cm^−1^): 3256, 3078, 2895, 1674, 1556, 1279, 1090. ^1^H NMR (400 MHz, DMSO-d_6_, *δ* ppm): 8.45 (s, 1H, NH), 8.21 (d, 2H, H-3′′, H-5′′), 7.81–7.73 (m, 4H, H-2′, H-3′, H-5′, H-6′), 7.47 (d, 2H, H-2′′, H-6′′), 5.61–5.53 (m, 1H, CH-3), 5.44 (s, 2H, CH_2_-2), 4.13 (s, 3H, CH_3_-4′), 2.78 (d, 3H, CH_3_-4). ^13^C NMR (DMSO-d_6_, *δ* ppm): 169.2 (C=O, NHCO), 161.8 (C, C-1′′), 142.9 (C, C-1′), 141.4 (C, C-4′), 139.9 (CH, C-3′′, 5′′), 126.4 (CH, C-3′, 5′), 123.8 (CH, C-2′, 6′), 116.2 (CH, C-1′′, 6′′), 114.8 (C, C-4′′), 68.6 (CH_2_, C-2), 51.3 (CH, C-3), 26.7 (CH_3_, C-4), 25.8 (CH_3_, CH_3_-C4′). mass: m/z 347 (M^+^), 348 (M+1, 18.2%), 349 (M+2, 99.8%), Anal. Calc. For C_17_H_18_BrNO_2_: C 58.63, H 5.21, N 4.02. Found: C 58.69, H 5.17, N 4.08. 

*(8) 2-(4-Nitrophenoxy)-N-(1-(p-tolyl)ethyl)acetamide (* **3h** *).* IR (KBr, cm^−1^): 3219, 3078, 2917, 1678, 1596, 1534, 1267, 1034. ^1^H NMR (400 MHz, DMSO-d_6_, *δ* ppm): 8.37 (d, 2H, H-3′′, H-5′′), 8.13 (s, 1H, NH), 7.92 (d, 2H, H-2′′, H-6′′), 7.79–7.53 (m, 4H, H-2′, H-3′, H-5′, H-6′), 6.78–6.57(m, 1H, CH-3), 5.30 (s, 2H, CH_2_-2), 3.39 (s, 3H, CH_3_-4′), 2.84 (d, 3H, CH_3_-4). ^13^C NMR (DMSO-d_6_, *δ* ppm): 168.4 (C=O, NHCO), 167.1 (C, C-1′′), 161.9 (C, C-4′′), 157.8 (C, C-1′), 139.6 (C, C-4′), 131.6 (CH, C-3′, 5′), 129.1 (CH, C-3′′, 5′′), 127.4 (CH, C-2′, 6′), 120.4 (CH, C-2′′, 6′′), 71.8 (CH_2_, C-2), 68.4 (CH, C-3), 44.6 (CH_3_, C-4), 41.9 (CH_3_, CH_3_-C4′). mass: m/z 314 (M^+^), 315 (M+1, 19.1%), 316 (M+2, 2.9%). Anal. Calc. For C_17_H_18_N_2_O_4_: C 64.96, H 5.77, N 8.91. Found: C 64.91, H 5.78, N 8.97. 

*(9) 2-(4-(Tert-butyl)phenoxy)-N-(1-(p-tolyl)ethyl)acetamide (* **3i** *).* IR (KBr, cm^−1^): 3243, 3056, 2978, 2943, 1635, 1575, 1266, 1044. ^1^H NMR (400 MHz, DMSO-d_6_, *δ* ppm): 8.51 (s, 1H, NH), 8.33 (d, 2H, H-3′′, H-5′′), 8.27–8.11 (m, 4H, H-2′, H-3′, H-5′, H-6′), 7.53 (d, 2H, H-2′′, H-6′′), 5.11–4.28 (m, 1H, CH-3), 4.37 (s, 2H, CH_2_-2), 3.81 (s, 3H, CH_3_-4′), 3.16 (d, 3H, CH_3_-4), 2.76 (s, 9H, (CH_3_)_3_).^13^C NMR (DMSO-d_6_, *δ* ppm): 170.8 (C=O, NHCO), 158.4 (C, C-1′′), 151.3 (C, C-4′′), 139.1 (C, C-1′), 138.2 (C, C-4′), 136.4 (CH, C-3′, 5′), 134.1 (CH, C-3′′, 5′′), 130.8 (CH, C-2′, 6′), 122.6 (CH, C-2′′, 6′′), 74.6 (CH_2_, C-2), 71.4 (CH, C-3), 51.7 (C, C-(CH_3_)_3_), 46.2 ((CH_3_)_3_), 38.1 (CH_3_, C-4), 36.7 (CH_3_, CH_3_-C4′). mass: m/z 325 (M^+^), 326 (M+1, 23.1%), 327 (M+2, 3.6%). Anal. Calc. For C_21_H_27_NO_2_: C 77.50, H 8.36, N 4.30. Found: C 77.43, H 8.40, N 4.36. 

*(10) 2-(4-Methoxyphenoxy)-N-(1-(p-tolyl)ethyl)acetamide (* **3j** *).* IR (KBr, cm^−1^): 3233, 2935, 2907, 2811, 1644, 1576, 1308, 1017. ^1^H NMR (400 MHz, DMSO-d_6_, *δ* ppm): 8.44 (s, 1H, NH), 8.21-8.13 (m, 4H, H-2′, H-3′, H-5′, H-6′), 7.69 (s, 4H, H-2′′, H-3′′, H-5′′, H-6′′), 5.75–5.61 (m, 1H, CH-3), 5.32 (s, 2H, CH_2_-2), 4.23 (s, 3H, OCH_3_), 2.78 (s, 3H, CH_3_-4′), 2.34 (d, 3H, CH_3_-4). ^13^C NMR (DMSO-d_6_, *δ* ppm): 171.2 (C=O, NHCO), 168.8 (C, C-4′′), 161.4 (C, C-1′′), 159.8 (C, C-1′), 158.4 (C, C-4′), 161.4 (C, C-1′′), 157.1 (CH, C-3′, 5′), 152.3 (CH, C-2′, 6′), 148.2 (CH, C-2′′, 3′′, 5′′, 6′′), 76.1 (CH_2_, C-2), 71.7 (CH_3_, OCH_3_), 65.4 (CH, C-3), 32.8 (CH_3_, C-4), 31.7 (CH_3_, CH_3_-C4′). mass: m/z 299 (M^+^), 300 (M+1, 19.2%), 301 (M+2, 2.8%). Anal. Calc. For C_18_H_21_NO_3_: C 72.22, H 7.07, N 4.68. Found: C 72.29, H 7.01, N 4.62.

### 2.3. Pharmacological Screening

#### 2.3.1. Animals

Wister albino rats of either gender weighing 140–180 g were obtained. The animals were divided into several groups of five animals each. All the animals were housed under standard ambient environment of temperature (25 ± 2°C) and relative humidity of 50 ± 5%. A 12 : 12 hour light : dark cycle was maintained. All the animals were allowed to have free access to water and standard palletized laboratory animal diet 12 hours prior to pharmacological studies. All the experimental procedures and protocols used in this study were reviewed and approved by the Institutional Animal Ethical Committee (IAEC).

#### 2.3.2. Preparation of Test Compounds

Test samples and the reference drugs were prepared as a suspension in 1% tween 80. Control group received 0.1 mL of tween 80 suspension orally. The second group (reference) received a dose of 50 mg/kg suspension of diclofenac sodium. Test groups were treated with a dose of 100 mg/kg of final synthesized compounds.

#### 2.3.3. Acute Toxicity

The acute toxicity study was carried out according to OECD guidelines [25]to found the successful dose of the test compounds after getting ethical clearance. Wister albino rats of either sex weighing between 140 and 180 g were divided into several groups of 5 animals each. Animals were starved for 12 hours prior to test. On the day of the experiment, animals were treated with different compounds to different groups in an increasing order of 10, 20, 100, 200, and 1000 mg/kg body weight orally. The animals were then observed continuously for 3 hours for common behavioral and autonomic profiles and then every 30 min for next 4 hours and finally for the next 24 hours or till death.

As per above toxicity test, it was observed that at a highest dose of 1000 mg/kg body weight, animals were found to be safe. But few changes were found in the behavioral reaction like touch response, alertness, and restlessness. Therefore, 1/10th of the highest tolerated dose, that is, 100 mg/kg body weight (b.w.), was chosen for the studies.

#### 2.3.4. Anticancer Activity

Compounds** 3a–j** were biologically evaluated for* in vitro* cytotoxicity using sulforhodamine B assay (SRB) based cellular protein content determination against two human cancer cell lines that contained MCF-7 (breast) and SK-N-SH (Neuroblastoma), respectively. Adriamycin drug was taken as positive control and the results are reported as percent control growth. In this current protocol, each cell line is preincubated on microtitre plate. Results for each test compound are reported as the percent growth of treated cells is compared with the untreated control cells. The compounds which reduce growth of cell lines to 32% or less are considered active.

#### 2.3.5. Anti-Inflammatory Activity

Carrageenan induced rat paw edema method [26] was employed for screening of the anti-inflammatory activity of the synthesized compounds listed in Table 1. The animals were divided into twelve groups of five each. One hour after oral administration of the drug, acute inflammation was produced by preparing aqueous suspension of carrageenan (1 %w/v, 0.1 mL) which was injected in the right hind paw in the subplanter region of each rat. A mark was applied on the leg at the malleolus to facilitate subsequent readings. The paw volume was measured plethysmometrically at 30 min, 2 hr, and 4 hr after the injection of carrageenan. The % inhibition was calculated by applying the Newbould formula [27]:

(1) $$\begin{array}{c} \%\text{Inhibition}=\left(1-\frac{V_{t}}{V_{c}}\right)\times 100, \end{array}$$

where *V*_*t*_ and *V*_*c*_ are the mean change in paw volume of treated and control rats, respectively.

#### 2.3.6. Analgesic Activity

The compounds exhibited an important analgesic activity as per Eddy's hot plate method [28]. Animals were individually placed on a hot plate maintained at a constant temperature (55°C) and the reaction of animals such as paw licking or jump response (whichever appears first) was taken as the end point. A cut-off time of 15 seconds was taken as maximum analgesic response to avoid any injury of the paws. The reference group was administered with a dose of 50 mg/kg of the suspension of diclofenac sodium (standard). The reaction time for each animal was noted on the hot plate at 30, 60, and 90 minutes after drug administration.

#### 2.3.7. Statistical Analysis

The results of anticancer, anti-inflammatory, and analgesic activities are shown in the Tables 2, 3, and 4, respectively. The results were expressed as mean ± SEM and were analyzed using one-way analysis of variance (ANOVA) followed by Dunnett's *t*-test. The probability of 0.05 or less was considered statistically significant. Statistical analysis was computed with the GraphPad Prism software version 5.01, GraphPad Software Inc. USA.

## 3. Results and Discussion

**Figure 2:**
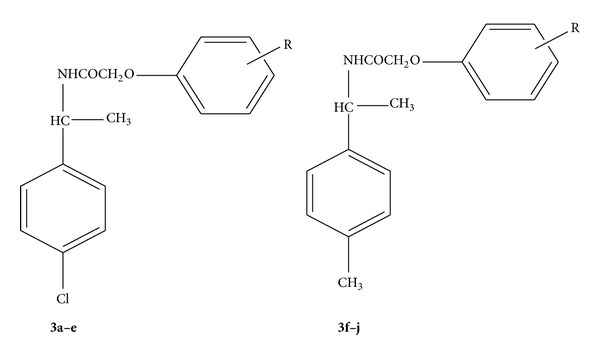
General structure of the synthesized compounds.

**Figure 3:**
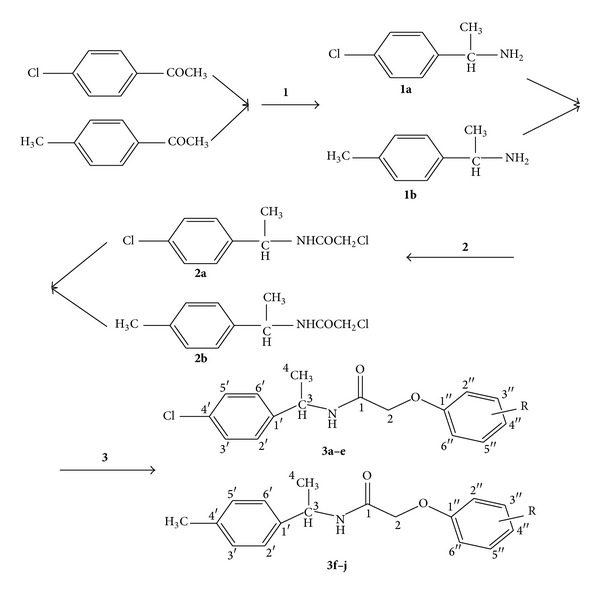
Synthesis of N-(1-(4-chlorophenyl)ethyl)-2-(substituted phenoxy)acetamide (**3a–e**) derivatives and 2-(substituted phenoxy)-N-(1-(p-tolyl)ethyl)acetamide (**3f–j**) derivatives.

A series of titled derivatives N-(1-(4-chlorophenyl)ethyl)-2-(substituted phenoxy)acetamide** 3(a–e)** derivatives and 2-(substituted phenoxy)-N-(1-(p-tolyl)ethyl)acetamide** 3(f–j)** derivatives (Figures 2 and 3) was synthesized as per scheme. 1-(4-chlorophenyl)ethanone and 1-(p-tolyl)ethanone were separately treated with ammonium carbonate and formic acid resulting in the formation of amines** 1a** and** 1b**, respectively. These amines on chloroacetylation with chloroacetyl chloride at 0°C in 10% sodium hydroxide medium give chloro compounds** 2a** and** 2b** which converted to (**3a–j**) by the reaction with different substituted phenols in presence of potassium iodide and potassium carbonate in dry acetone as a solvent. The structure of newly synthesized compounds (Figures 2 and 3) was confirmed by spectral data (IR, ^1^H NMR, ^13^C-NMR and mass); Table 1 shows the physical data of compounds (**3a–j**).

Further IR spectrum of compounds (**3a–j**) showed characteristic absorption bands at range of 3205–3266 cm^−1^ were attributed to NH, 1635–1711 cm^−1^ accounting for C=O of amide group and 1533–1644 for C=C in the aromatic ring. Two peaks at range of 1200–1335 and range of 1017–1235 indicate the presence of C–O–C linkage. The structure of compounds was further supported by mass spectral data.

**Figure 4:**
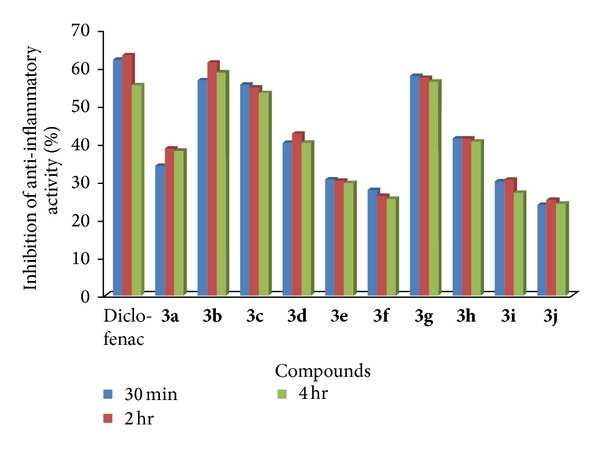
Graphical representation of (% inhibition) of anti-inflammatory activity.

**Figure 5:**
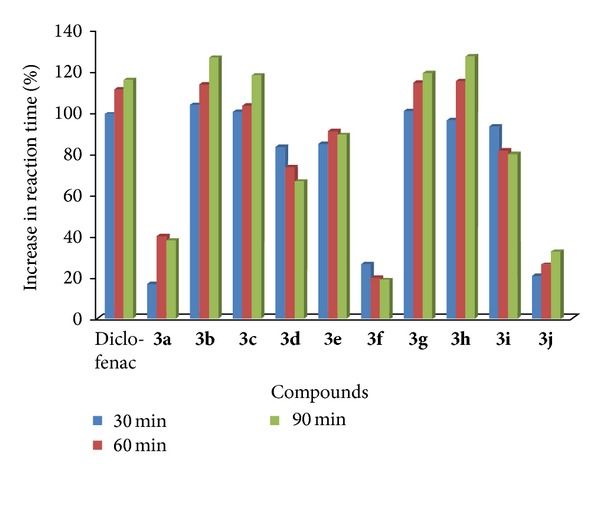
Graphical representation of % increase in reaction time for analgesic activity.

All compounds** 3a–j** were subjected for preliminary toxicity test as per organization for Economic Co-operation and Development (OECD) guidelines in rats and 100 mg/kg was used as therapeutic dose. Acute anti-inflammatory activity was performed by carrageenan induced rat paw edema method by Newbould [26]. Diclofenac sodium was used as a reference standard. Compounds** 3b**,** 3c**, and** 3g** exhibited potent anti-inflammatory activity similar to the standard (Table 3, Figure 4). The analgesic effects of compounds** 3b**,** 3c**,** 3e**,** 3g**, and** 3h** were found to be nearly of standard (Table 4, Figure 5).

Compounds** 3c** were found to possess anticancer, anti-inflammatory, and analgesic activities nearly to the standard because of the presence of NO_2_ group and Br groups at position 4 of the phenoxy ring.

From the comprehensive analysis of the results in current studies, we conclude that synthesized compounds have anticancer, anti-inflammatory, and analgesic activities because of the presence of 1-phenylethylamine as basic ring. In analysis of these observations, we conclude that this series (**3a–j**) could be developed and explored as a novel class of anticancer and NSAIDs. However, further detailed pharmacological program is required to recognize the potent molecule without various side effects.

### 3.1. Structure Activity Relationship (SAR)

By comparing the cytotoxic potency of newly synthesized compounds** 3a–j**, a few inferences could be drawn as follows. (i) The presence of tert-butyl, methoxy, and nitro substituent at 4 positions does not contribute towards cytotoxicity as can be seen in compounds** 3d**,** 3j**, and** 3h** which showed poor growth inhibition against both of the cancer cell lines used. (ii) The presence of Br group at 4 positions contributes significantly towards the enhancement of inhibitory activity. (iii) The presence of NO_2_ group at 4 positions in the phenoxy nucleus attached to 1-(4-chlorophenyl)ethanamine makes the compound potent antiproliferative agent with dual inhibition of both MCF-7 and SK-N-SH cell lines.

After carrying out the anti-inflammatory and analgesic screenings of synthesized compounds, the following conclusions are drawn. (i) Presence of Br and NO_2_ groups at 4 positions in the phenoxy nucleus contributes towards anti-inflammatory activity. (ii) Presence of tert-butyl and methoxy substituent at 4 positions does not contribute towards anti-inflammatory activity. It is also observed that compound** 3c** with nitro substituent in the 4 positions of phenoxy nucleus contributed effectively towards anticancer, anti-inflammatory, and analgesic activities. The analgesic activities may be due to inhibition of COX/LOX pathways which are the possible mechanisms of induction of pain. In our future research programme, these pathways which are highly active in malignant cancerous growth would be targeted. This research would be a new breakthrough in the area of oncology where enzyme mediators and COX-2 and LOX-2 inhibitors would be utilized in suppression of tumor growth.

*Scheme I.* Synthesis of N-(1-(4-chlorophenyl)ethyl)-2-(substituted phenoxy)acetamide derivatives** (3a–e)** and 2-(substituted phenoxy)-N-(1-(p-tolyl)ethyl)acetamide derivatives** (3f–j)**. Reagents and conditions:** (1)** ammonium carbonate and formic acid, heat very slowly till 165°C→Ketone, Heat, 4-5 h, 180–185°C→Reflux, concentrated hydrochloric acid, 2-3 hour, water bath→extraction, diethylether→Strongly alkaline, 30% sodium hydroxide→extraction; Diethylether** (2)** Chloroacetyl chloride, 10% sodium hydroxide, ice bath, pH 9-10.** (3)** Substituted phenols, dry acetone, Potassium Carbonate, Potassium Iodide, Reflux.

In the series of ten compounds for activity against MCF-7 (breast) cell line, compounds** 3b** and** 3c** showed active inhibition of the cancer cells. These two compounds were found to be highly active against the cancer cell lines. Compounds** 3d**,** 3h,** and** 3i** showed mild to moderate cytotoxic activities, whereas the remaining compounds were found to be completely inactive.

Out of ten compounds,** 3f** and** 3c** were found to be modestly active against SK-N-SH (neuroblastoma) cell line, and the remaining compounds were found to be inactive.

## Figures and Tables

**Table 1:** Characterization data of N-(1-(4-chlorophenyl)ethyl)-2-(Substituted phenoxy)acetamide derivatives (**3a**–**e**) and 2-(substituted phenoxy)-N-(1-(p-tolyl)ethyl)acetamide derivatives (**3f**–**j**).

Compound	R	Yield (%)	Melting point∗ (°C)	Rf value^#^	Molecular formula
**3a**	H	61.4	171–173	0.37	C_16_H_16_ClNO_2_
**3b**	4-Br	58.3	154–156	0.45	C_16_H_15_BrClNO_2_
**3c**	4-NO_2_	59.1	165–167	0.41	C_16_H_15_ClN_2_O_4_
**3d**	4-C-(CH_3_)_3_	62.7	205–207	0.50	C_20_H_24_ClNO_2_
**3e**	4-OCH_3_	71.0	126–128	0.38	C_17_H_18_ClNO_3_
**3f**	H	67.8	154–156	0.32	C_17_H_19_NO_2_
**3g**	4-Br	57.2	189–191	0.46	C_17_H_18_BrNO_2_
**3h**	4-NO_2_	74.5	148–150	0.51	C_17_H_18_N_2_O_4_
**3i**	4-C-(CH_3_)_3_	65.9	172–174	0.39	C_21_H_27_NO_2_
**3j**	4-OCH_3_	70.4	142–144	0.47	C_18_H_21_NO_3_

∗Recrystallization with ethanol. ^#^Stationary phase: silica gel; mobile phase: n-hexane : ethyl acetate (1 : 1); iodine vapors as visualizing agent.

**Table 2:** Result of anticancer activity.

Compound	% Control growth		Activity
	MCF-7 (breast)	SK-N-SH (neuroblastoma)	
**3a**	88.9	88.2	Inactive
3b*	30.5	67.6	Active
3c***	31.6	32.3	Active
**3d**	54.0	72.1	Inactive
**3e**	88.0	95.7	Inactive
3f***	71.9	32.1	Active
**3g**	44.4	69.1	Inactive
**3h**	70.0	71.8	Inactive
**3i**	58.3	73.1	Inactive
**3j**	74.0	80.1	Inactive

∗Active against MCF-7 (breast) cell line.

∗∗Active against SK-N-SH (neuroblastoma) cell line.

∗∗∗Active against both MCF-7 (breast) and SK-N-SH (neuroblastoma) cell lines.

Growth percentages less than 32 are considered as active.

**Table 3:** Result of anti-inflammatory activity.

Compound	Mean changes in paw edema (mL) mean ± SEM			% Inhibition		
	30 min	2 hr	4 hr	30 min	2 hr	4 hr
Control	0.612 ± 0.014	0.754 ± 0.051	0.621 ± 0.013	—	—	—

Diclofenac sodium	0.232 ± 0.021	0.277 ± 0.019	0.277 ± 0.027	62.09 ± 0.095	63.26 ± 0.095	55.39 ± 0.095

3a**	0.403 ± 0.024	0.462 ± 0.026	0.384 ± 0.013	34.15 ± 0.011	38.72 ± 0.010	38.16 ± 0.019

3b**	0.265 ± 0.033	0.292 ± 0.05	0.256 ± 0.020	56.69 ± 0.033	61.27 ± 0.050	58.77 ± 0.039

3c*	0.272 ± 0.009	0.341 ± 0.041	0.290 ± 0.030	55.55 ± 0.041	54.77 ± 0.023	53.30 ± 0.013

3d**	0.366 ± 0.043	0.433 ± 0.009	0.371 ± 0.010	40.19 ± 0.014	42.57 ± 0.063	40.25 ± 0.019

3e*	0.425 ± 0.013	0.526 ± 0.019	0.437 ± 0.011	30.55 ± 0.033	30.23 ± 0.011	29.62 ± 0.024

3f*	0.442 ± 0.039	0.556 ± 0.103	0.463 ± 0.019	27.77 ± 0.134	26.25 ± 0.028	25.44 ± 0.020

3g**	0.258 ± 0.024	0.322 ± 0.09	0.271 ± 0.17	57.84 ± 0.006	57.29 ± 0.008	56.36 ± 0.04

3h*	0.359 ± 0.009	0.442 ± 0.009	0.369 ± 0.024	41.33 ± 0.039	41.37 ± 0.020	40.57 ± 0.024

3i**	0.428 ± 0.020	0.524 ± 0.024	0.453 ± 0.07	30.06 ± 0.041	30.50 ± 0.09	27.05 ± 0.009

3j*	0.466 ± 0.041	0.564 ± 0.061	0.471 ± 0.011	23.85 ± 0.043	25.19 ± 0.041	24.15 ± 0.011

*P < 0.05 significant from control.

**P < 0.01 significant from control.

**Table 4:** Result of analgesic activity.

Compound	Reaction time (S) after drug administration (mean ± SEM)			% Inhibition		
	30 min	60 min	90 min	30 min	60 min	90 min
Control	2.67 ± 0.020	2.69 ± 0.025	2.70 ± 0.03	—	—	—

Diclofenac sodium	5.31 ± 0.03	5.67 ± 0.019	5.82 ± 0.014	98.87 ± 0.214	110.78 ± 0.217	115.55 ± 1.620

3a*	3.11 ± .004	3.76 ± 0.019	3.72 ± 0.07	16.47 ± 0.019	39.77 ± 0.033	37.77 ± 0.041

3b**	5.43 ± 0.019	5.74 ± 0.033	6.11 ± 0.010	103.37 ± 0.043	113.38 ± 0.027	126.29 ± 0.90

3c*	5.34 ± 0.041	5.46 ± 0.011	5.88 ± 0.028	100 ± 0.006	102.97 ± 0.011	117.77 ± 0.081

3d**	4.89 ± 0.043	4.66 ± 0.010	4.49 ± 0.041	83.14 ± 0.071	73.23 ± 0.063	66.29 ± 0.038

3e**	4.92 ± 0.011	5.13 ± 0.020	5.10 ± 0.063	84.26 ± 0.023	90.70 ± 0.006	88.88 ± 0.075

3f*	3.37 ± 0.05	3.22 ± 0.008	3.20 ± 0.019	26.21 ± 0.014	19.70 ± 0.023	18.51 ± 0.010

3g**	5.35 ± 0.023	5.76 ± 0.09	5.91 ± 0.020	100.37 ± 0.041	114.12 ± 0.020	118.88 ± 0.61

3h**	5.23 ± 0.019	5.78 ± 0.063	6.13 ± 0.011	95.88 ± 0.05	114.86 ± 0.039	127.03 ± 0.071

3i**	5.15 ± 0.010	4.88 ± 0.014	4.85 ± 0.028	92.88 ± 0.023	81.41 ± 0.043	79.62 ± 0.91

3j*	3.22 ± 0.020	3.39 ± 0.041	3.57 ± 0.019	20.59 ± 0.008	26.02 ± 0.041	32.22 ± 0.38

*P < 0.05 significant from control.

**P < 0.01 significant from control.
